# Burnei’s procedure in the treatment of long bone pseudarthrosis in patients having osteogenesis imperfecta or congenital pseudarthrosis of tibia – preliminary report

**Published:** 2012-06-18

**Authors:** C Vlad, I Georgescu, TS Gavriliu, DI Hodorogea, T El Nayef, D Dan

**Affiliations:** “Maria Sklodowska Curie” Children Emergency Hospital, Bucharest, Romania

**Keywords:** bone graft, osteogenesis imperfecta, congenital pseudarthrosis of tibia, large bone defect, circumferential compression

## Abstract

**Rationale:** given the recalcitrant behaviour of pseudarthrosis in osteogenesis imperfecta (OI) and congenital pseudarthrosis of the tibia (CPT), there is no ideal solution to treat such challenging deformities. The reconsideration of the already known principles, by using the modern technology, may generate new treatment methods.

**Aim:** the present paper presents the preliminary results of an original reconstruction procedure used to treat large bone defects in paediatric orthopaedics. A case series study, the surgical technique, complications and illustrative cases are presented.

**Methods and results:** 3 cases of pseudarthrosis in OI and 2 cases of CPT were operated by using this technique. The principles of the method are to create an optimal osteoconductive and osteoinductive environment by using a bone autograft, bone allograft and bone graft substitutes and to provide a good stabilisation of the bones. We operated 3 patients with OI and 2 patients with CPT. Four patients had multiple previous surgeries. The follow-up period ranged from 3 to 28 months. Four of the five patients are able to ambulate independently at the moment this paper was written.

**Discussion:** we believe that the present technique could be a reliable alternative to other procedures, especially in cases of repeated failures.

## Introduction

The surgical treatment of pseudarthrosis includes many procedures, the goal being the improvement of the surgical technique, the reduction of immobilisation period, the decrease of the intraoperative incidents and accidents and the decrease of the complications rate, especially the recurrence.

Large bone defects are challenging entities in paediatric orthopaedics. The same procedure was used in our department for the treatment of long bone pseudarthrosis in children having congenital pseudarthrosis of tibia (CPT) or osteogenesis imperfecta (OI), two different entities in which large bone defects may appear. According to the severity of CPT, amputation may be advocated by one [**1**]. On the other hand, the inability to walk with or without an assisting device is the natural course of the disease in many forms of OI [**2**].

Congenital pseudarthrosis of the tibia is one of the most redoubtable diseases in paediatric orthopaedics. According to some authors [**3**], 10% of all CPT will not achieve the bone fusion. Osteogenesis imperfecta is a genetic disorder altering the type I collagen formation, generating long bones deformations including pseudarthrosis with large bone defects. 

The main problems in such diseases are the following: to provide a good splint of the operated bone, to achieve the bone union, to augment the bone mass.

Amputation, the last treatment option in severe forms or recurrent fracture in CTP was considerably diminished according to many authors or should be excluded by most of them [**4**]. Traditional methods used in the treatment of CPT have at least one disadvantage: Ilizarov method and pedicled fibular flap method do not provide enough bony mass; Charnley-Williams technique does not provide a good enough splint. In OI, the bones are long, curved and thin. The techniques of bone rodding in OI focus on splinting bones without enhancing the bone mass. 

The technique described in this paper tries to provide solutions for the three main problems pointed out above; the present paper is a case series study presenting short-term results. Radiological and functional data, posture and locomotion parameters were evaluated.

## Materials and methods

We used the same complex technique of bone reconstruction in 5 patients having CPT or OI. The principles of the technique consist of creating an optimal osteoconductive and osteoinductive environment with a good internal stabilisation of the affected limb. The osteoconduction was provided by the massive allograft: fresh frozen ribs or fibula, dried femoral shaft. The osteoinductive effect was enhanced by the DBM (demineralised bone matrix) in the allograft bone substitute. The internal fixation has a double effect: to splint the operated bone on the entire length and to compress the bone allograft circumferentially by using wires or cables and reconstruction plates.

**Fig. 1:**
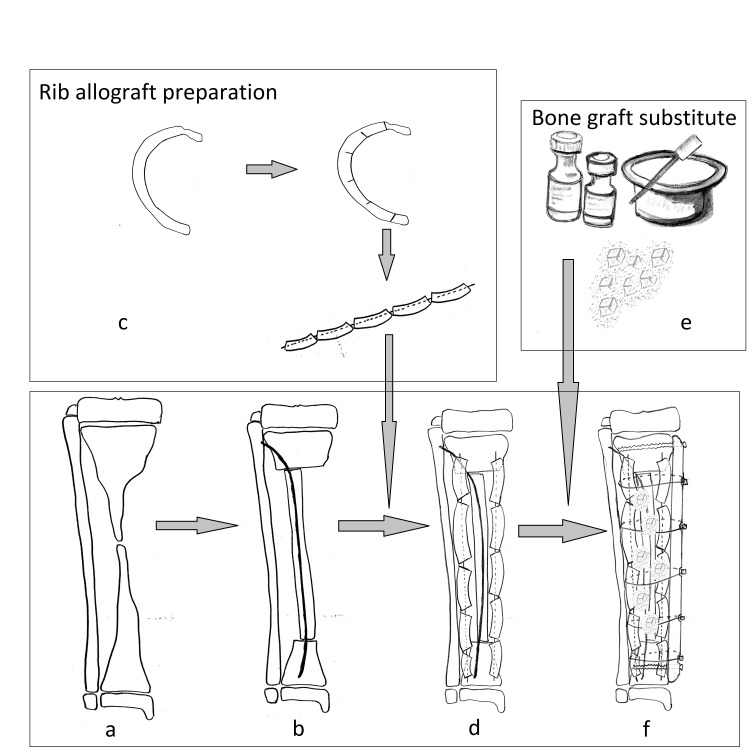
Scheme of the Burnei’s procedure. (a) a tibia affected by pseudarthrosis; (b) central core composed of a bone allograft splinted with a K wire or a titanium elastic nail; (c) the rib allograft is incompletely sectioned, on one third of its width, at intervals of 2-3 cm, afterward the rib is reshaped on a K wire; (d) rib allografts are placed in an onlay fashion, the extremities of the K wires are pinned in the metaphyses, proximally and distally; (e) the bone graft substitute is prepared; (f) the free spaces are filled with bone graft substitute; if needed, 2-3 allograft bars are added; the circular compression is performed with cables on a reconstruction plate.

In large pseudarthrosis, the surgical steps are the following:

-step 1: bone ends resection and construction of a central bone axis, ideally by using a bone autograft, splinted with an elastic nail or a Kirschner wire;

-step 2: onlay application of rib bone allograft;

-step 3: addition of bone substitute containing DBM;

-step 4: addition of 2 or 3 cortical allograft bars which overlay the healthy bone extremities on 2-3 cm;

-step 5: circumferential compression of bone graft on a reconstruction plate with cables or wires;

In cases with close pseudarthrosis, the first step was not performed. In growing patients, the physis must be preserved. Three layers of bone graft can be identified in the composite construction described above (see Fig. 1). Ideally, the central core must be composed of a splinted autograft, with the dimensions compatible with the normal diaphysis, allograft (bone bank rib or fibula graft) may be used. The splint can be a K wire or a titanium elastic nail. The second layer is composed of a bone graft substitute containing osteoinductive substances and by 2-4 rib allografts prepared in a special manner: the rib is sectioned at 3-5 levels and a K wire is passed through all fragments, the final shape of the rib being straight (**see Fig. 1**). The third layer is made up of 2-3 cortical allograft bars. The fragments are obtained by longitudinal split of a bone bank diaphysis allograft in 4-6 fragments. Finally, all bone grafts are circumferentially compressed by cables or wires alongside a reconstruction plate.

Between September 2009 and September 2011, 6 segments in 5 patients were operated in our institution. The patients presented the following conditions: 1 large CPT, 1 close CPT, 1 large pseudarthrosis of tibia in a patient having OI, 1 femur incurvation in the same patient, 1 close pseudarthrosis in a patient having OI, 1 tibia curvature in a patient having OI, see **Table 1**.

**Table 1:** The patients’ conditions

Case	Age at first visit	Sex	Age at last follow-up	Operated segments	Personal history	Main diagnostic	Pseudarthrosis type (large or close)	Surgery type	Complications	Bone union	Full-weight bearing of operated limb	Orthosis	Residual deformity	Follow-up period
1.	5y6mo	M	10y	Left tibia	y6m bone transport Ilizarov method 6y8m fixator removal	CPT Crawford IV,, NF1	large	8y 9m allograft-dall miles plate cm k wire 9y plate and cables removal	Site infection which needed drainage and resuture	N	N	Y	-	1y
2.	12y	F	12y7m	Right tibia	-	CPT Crawford IV, NF1	close	12y7m LCP plate, steel wires, Intramedullary splint	Site infection requiring multiple debridement and wound healing per secundam	Y	4m after surgery	Y	N	1y6m
3.	20y	F	28y3m	Right femora	20 y: Sofield Millar osteotomies and splinting of right femora and tibia with sheffield expandable rods; femoral rod broke 6 years later	OI sillence III	Two levels, close	27y3m:Fibular, femoral and splinted rib, allograft, Allomatrix, Dall-Miles plate, centromedullary splint	N	Y	11months after surgery with crutches	Y	Mild coxa vara	1y
4.	2y	M	10y4my	Left tibia	4y Expandable rodding of left tibia 6y expandable rods of right femora	OI sillence IV	Bowed tibia	10y Rigth tibia: soffield-Millar osteotomies, intramedullary osteosynthesis with TEN, rib allograft, femoral allograft Allomatrix, Dall Miles plate and cables	Superficial hematoma	Y	N	Y	N	3m
5.	21y6m	F	27y	Right tibia Left femora	14 y: Rush rods of both femoras 18 y:Fibular maternal allograft for both tibias fixated by plate end screws	OI sillence III	Large pseudarthrosis on tibia Bowed left femora	24y Right tibia: fibular allograft, splinted rib allograft, intramedullary splint, Dall Miles plate and cables periosteal substitute 25y6m Left femora: Dall Miles plate and cables, allograft, graft jacket 26y: right tibia: plate and cables removal	N	Y	Right tibia :10 months after surgery Left femora: 12 months after surgery, with cruthes	Y	N	2y4m for right tibia 1y for left femora

## Results 

The patients’ age at the moment of surgery ranged from 8 years and 9 months to 27 years and 3 months with an average of 17 years and 10 months. The average follow-up period was of 14 months, ranging from 3 months to 28 months. The patients were 3 females and 2 males.

All the surgeries were performed in our department, under general anaesthesia. All the patients except for patient number 2 (see **Table 1**) had previous multiple surgeries. Surgical approach was designed to avoid skin necrosis.

The patients were mobilized on the third day postoperatively in cases with a good function of the contralateral pelvic limb, patient 1 and patient 2 (see **Table 1**). In other cases, the patients were mobilized at three months after surgery. Plaster cast immobilisation after the surgery was used in two cases; patient 4 and patient 5 (see **Table 1**). Full weight bearing on operated limb was possible between 4 and 12 months after the surgery, an average of 9 months. Independent walk was achieved in 3 patients. One patient (case 2) is able to walk and run.

Bone union was obtained in 5 of 6 operated segments. Three patients were able to fully bear the weight of the operated segments. Patient number 4 (see **Table 1**) was allowed to progressively bear the weight of the operated segment. Patient number 1 (see **Table 1**) has not achieved a good quality bone fusion at the time of writing this manuscript. 

The residual deformity, a mild coxa vara, was present in patient 3, operated for two levelled pseudarthrosis in osteogenesis imperfecta. Patient number 4, having osteogenesis imperfecta, presented a 2 cm limb length discrepancy due to a coxa vara deformity.

The bone shape was restored in all the cases. The X-rays exam after 1 year showed the bone graft integration and allowed the plate removal in case number 5 (see **Table 1**); at 2 years, the bone structure was reconfigured in a centripetal way.

Complications were noted in two cases. No complication compromising the final result was noted. Case number 2 presented a superficial infection and healed per secundam intentionem. Case number 1 presented a skin fistula. After three months of evolution, the decision to perform the surgical circumferential debridement of the wound was made. A suction drain was placed and the wound healed after 14 days. The new formed bone corresponding to the fistula presented a low-density image on X-ray, of about 4 cm in length. The demineralisation process was accompanied by the mobilisation of a K wire, which protruded at a subtegument level, at the heel. The K wire was explored surgically, the demineralised zone was found to be mobile and we decided to obdurate it with Prodense® (Wright medical Technology, Inc., USA).

Illustrative case reports

**Fig. 2:**
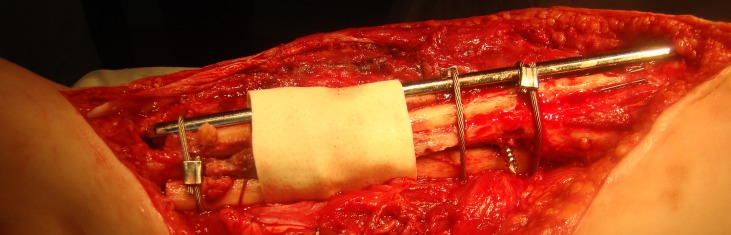
Intraoperative aspect of a tibia operated with the Burnei’s technique

Case number 2 (see **Table 1**) is a 12-year-old girl having a Crawford IV CPT of right tibia (**Fig. 2**). 

The bone extremities were cleaned of fibrous tissue, massive bone allograft was added, filling the gap and bridging the bone defect. A titanium elastic nail was used as an intramedullary splint. A plate with wires was used. The case was complicated by a superficial site infection requiring multiple debridements, the wound being healed per secundam intentionem. Four months after the surgery, the fully bore patient, weighted on the operated limb. At 1 year and a half follow-up period, the patient was able to walk and run. The radiograms (Fig. **3a,3b**) show a good quality callus formation that bridges the bone defect.

**Fig. 3a:**
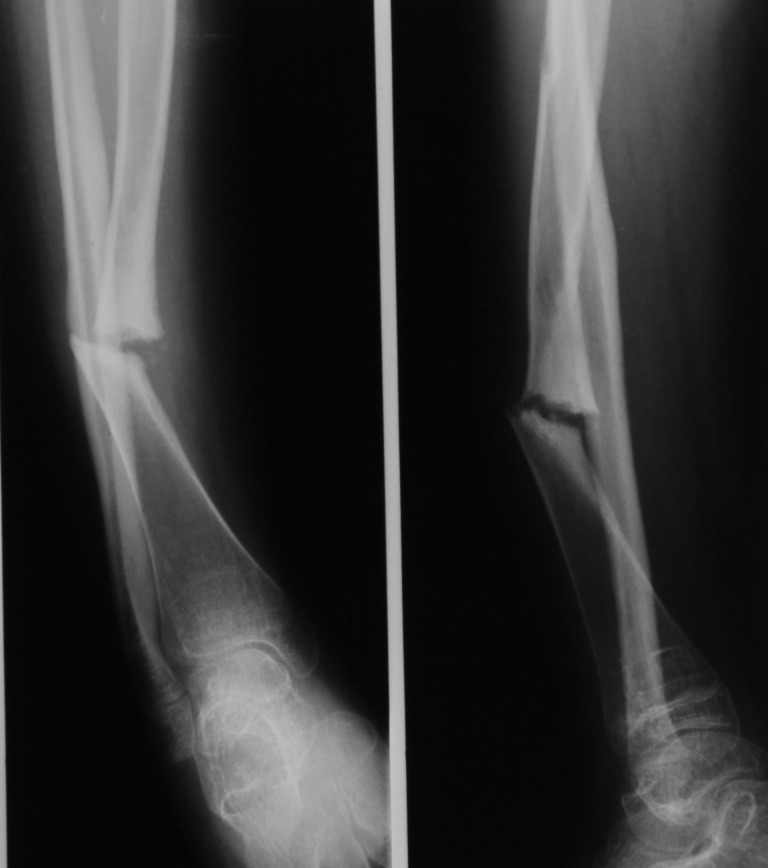
Preoperative X-ray image of case 2, twelve-year-old girl presenting a congenital pseudarthrosis of right tibia, Crawford IV.

**Fig. 3b:**
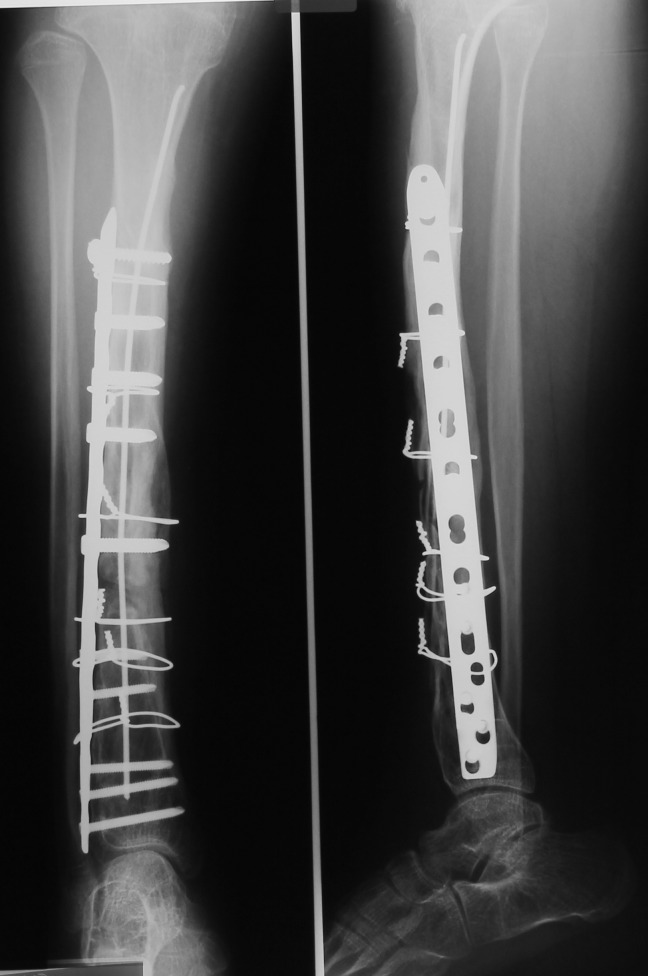
Case number 2, postoperative radiogram at 1 year and 6 months follow-up period. The callus bridges and fills the bone defect. The fixation devices are correctly

Case number 5 (see **Table 1**) is a 21-year-old female suffering from type III osteogenesis imperfecta. At the age of 14, both femurs were splinted with Rush rods. At the age of 18, both tibias were grafted with a maternal fibular allograft. Both surgeries were performed in another institution. After 2 years, the grafts were absorbed and the right tibia developed a mobile pseudarthrosis (**Fig. 4a**). 

**Fig. 4a:**
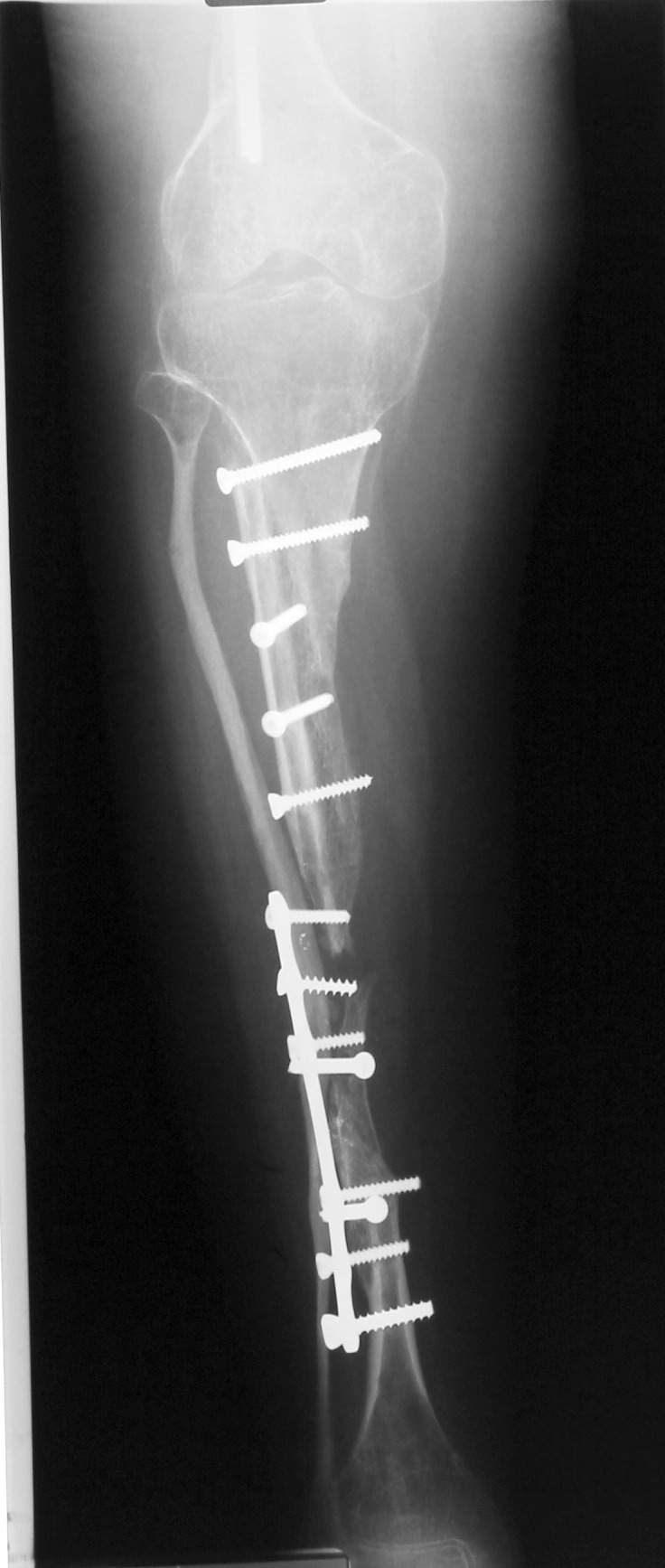
Case number 5, the fibular graft added to the right tibia was absorbed

At the age of 24, we performed the complex reconstruction by using bone bank fibular allograft, rib allografts, a periosteal substitute (Graftjacket®, Wright medical Technology, Inc., USA) and a containing DBM bone substitute (Allomatrix®, Wright medical Technology, Inc., USA). The rib allografts were reshaped over a small K wire. The allograft was circumferentially compressed with cables against a Dall-Miles plate® (Stryker Corporate, USA). Kischner wires were used to provide an intramedullary splint (**Fig. 4b**). 

**Fig. 4b:**
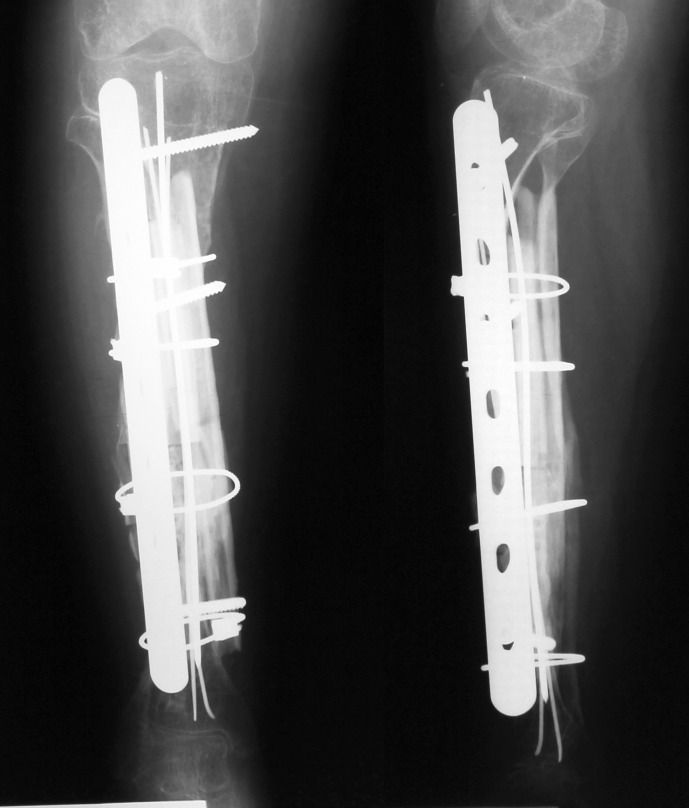
Case number 5, one year after the surgery, the radiogram showed good allograft integration

The wound healed per primam intentionem. Two years after the surgery, the plate and cables were removed (**Fig. 4c**). Proper integration of the bone graft is seen on radiograms.

**Fig. 4c:**
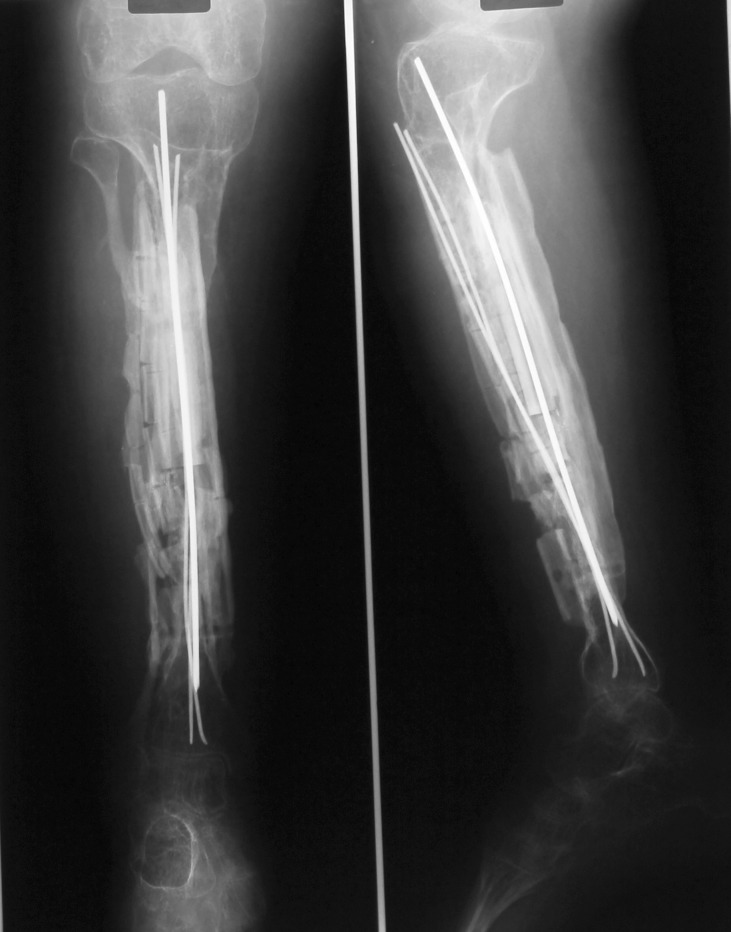
Case number 5, the plate and cables were removed two years after the surgery

## Discussions

The non-union recurrence, once, twice or even thrice, after the treatment by using an external circular frame, or the severe forms of pseudarthrosis, with thin bones or large bone defects, determined this new approach, especially for those children who rejected the amputation or a second or third use of the external fixator.

We already reported the use of longitudinal splitting and lateral traction technique in patients having osteogenesis imperfecta [**2**]. However, the width of the bone shafts does not increase adequately. Bone metabolism in osteogenesis imperfecta is characterized by an increased turnover. One may speculate that the increased turnover will allow a faster integration of the bone allograft.

Recent or neglected cases of pseudarthrosis in OI or type 1 neurofibromatosis (NF1), which presented bone bowing, thin bones or axial deviations, received the same treatment: massive bone allograft and bone substitute, circumferential compression on plate, with or without central splint, with or without periosteal membrane substitute.

According to a study performed in 2000 among the members of the European Paediatric Orthopaedic Society, the use of Ilizarov external circular frame reduced the failure rate at 25% [**5**]. Onishi [**3**] reported a fusion rate of 84% for the use of Ilizarov method in CPT in a Japanese study. Paley [**6**] reported a 94% fusion rate after one procedure, 100% fusion rate after 2 procedures but a refractor rate in 5 cases of 16 operated tibias (31%). One of the criticism regarding the Ilizarov technique is that the pseudarthrosis ends remained avascular after the transport [**7**].

During the last years, the orthopaedic surgeons agreed [**7**] that the aggressive bone grafting should be expected in such cases. The main problem after obtaining the fusion was the re-fracturing at the pseudarthrosis site [**6**].The technique described above tries to solve this problem by providing massive bone allograft and good fixation. 

The success of the surgery lays in the details. A compact tubular allograft will take too long to be incorporated and remodelled. The solution is to use many bone struts in order to allow the osteoconduction on a larger surface. The risk of dead space development is avoided by graft compression with the help of cables against a plate. In the same time, the plate will provide a good stabilisation of the bones.

During the last year, many bone graft products have become widely used in paediatric orthopaedics [**8**]. The massive allograft addition should be done quantum sufficit, in order to allow the skin closure. Evaluation is based on soft tissues elasticity quantity of the bone graft, which may be well covered by the soft tissues. Too much tension during the wound closure will increase the risk of wound dehiscence. 

In extreme situations, the massive bone graft, bone substitutes and circumferential graft compression could be a reliable solution for the deceived and depressed patients and parents, in order to avoid amputation.

**Acknowledgements**

Professor Gh. Burnei operated all the patients in the present study.

**Sources of Funding**

This paper is partly supported by the Sectorial Operational Programme Human Resources Development (SOPHRD), financed from the European Social Fund and by the Romanian Government under the contract number POSDRU 64331.
